# Using mobile phone Re-call notification as a primary care practice to improve vaccination uptake for children under five in baghdad, Iraq, 2022

**DOI:** 10.1177/22799036261470482

**Published:** 2026-07-17

**Authors:** Firas Jabbar, Kamal A. Kadhim, Riyadh A. Alhilfi, Faris Lami, Abdulaal Chitheer, Salman Rawaf, Celine Tabche, Mona Kuroiwa, Helene Davies, Alaa Rahi

**Affiliations:** 1 Department of EPI, Public Health Directorate, Ministry of Health, Baghdad, Iraq; 2 Public Health Directorate, Ministry of Health, Baghdad, Iraq; 3 Baghdad Medical College, Ministry of Higher Education, Iraq; 4 WHO Collaborating Centre for Public Health Education and Training, School of Public Health, 4615 Imperial College London, UK; 5 17096 Unicef, Health and Nutrition Section, Baghdad, Iraq

**Keywords:** reminder/re-call notification, immunization, vaccine, coverage, Iraq, primary care

## Abstract

**Introduction:**

Reminder and recall (RR) interventions are proven strategies for improving childhood immunization coverage. However, evidence on their feasibility and effectiveness in routine primary healthcare settings in Iraq is limited. This study evaluated a direct, person-to-person mobile phone recall intervention to improve vaccine uptake among children under five years of age in Baghdad Province in 2022.

**Methods:**

The intervention was implemented in 50 Primary Healthcare Centers (PHCs) with the lowest routine immunization performance, including 20 centers in Karkh and 30 in Rusafa. Eligible participants were children under five years of age who were overdue for one or more vaccines in the national Expanded Programme on Immunization (EPI) schedule, excluding Bacillus Calmette–Guérin (BCG) vaccine administered at birth. Trained EPI staff contacted caregivers through direct telephone calls, with up to six biweekly attempts over a three-month period. Standardized data collection forms were used under supervision of the Directorate of Public Health.

**Results:**

A total of 17,241 children (47.8% female) with 48,919 overdue vaccine doses were identified, of whom 15,087 (87.4%) had valid mobile phone numbers. Among these, 28,130 call attempts resulted in 10,061 caregiver responses. Most respondents (87.5%) agreed to schedule vaccination appointments. Ultimately, 6,831 children attended PHCs and received 13,386 overdue vaccine doses, corresponding to 39.4% child-level and 27.4% dose-level coverage among targeted defaulters.

**Conclusion:**

Direct cellphone-based recall interventions are feasible and operationally effective for improving routine childhood immunization in Iraq. As a low-cost strategy embedded within existing EPI structures, this approach has strong potential for scale-up in similar settings.

## Introduction

Routine vaccination against vaccine-preventable diseases (VPDs) is a cost-effective intervention that fundamentally reduces morbidity and mortality, especially for children. However, low and middle-income countries (LMIC) have low vaccination uptake and completion rates.^1^ In Iraq, despite substantial improvements in recent years, the national vaccine coverage in 2021 for children under five has stalled at about 75% for the measles vaccine and 78% for Pentavalent 3. These levels remain well below the recommended targets of at least 95% coverage for measles^2^ and 90% national coverage for Pentavalent 3,^3^ with coverage remaining far from optimal (95% for measles and 90% for Penta 3) in many health districts.^4^

Moreover, the COVID-19 pandemic has had a profound impact on routine immunization (RI) services provided at PHCs due to a shortage in health staff as increasing demand at COVID-19 management centers, high infection rates and long isolation periods, as well as caregivers’ access difficulty due to containment measures. Distribution of COVID-19 vaccines at the Primary Healthcare Center (PHC) level also resulted in less time allocated for health staff to implement outreach activities.^5^

Various community-based factors affect vaccine uptake, including decreased demand for vaccination, limited awareness of its importance, and restricted access to health facilities. These factors are associated with low coverage^6–9^ and high dropout rates between doses, particularly for vaccines scheduled later in the program.^10^ Caregivers may forget, lose interest, or be too busy to attend scheduled appointments. Planned reminders for caregivers may therefore play an important role in ensuring timely vaccination.^11^

It has been identified that there is a significant need to expand access to information and communication technologies to achieve sustainable development by 2030. Such technologies can potentially address and measure progress toward several Sustainable Development Goals.^12^

Growingly, digital technologies are becoming an important resource for health services delivery and public health. Mobile wireless technologies are particularly relevant because of their ease of use, broad reach, and wide acceptance. According to the World Bank-reported estimates, global mobile cellular subscriptions exceeded 9.17 billion mobile cellular subscriptions.^13^ Separate estimates indicate that more than 70% of these subscriptions are located in LMICs.^14^ For instance, in 2021, Iraq was ranked 165^th^ in global cellphone usage, with approximately 86 subscriptions per 100 citizens.^12^

Reminder and recall (RR) interventions are usually used to remind members of a target population that vaccinations are due (reminders) or late (re-call). RR differ in content and are tailored for individual clients, including educational messages about vaccination importance. There are various methods to deliver RR notices like telephone, letter, postcard, and text messages.^15^

Ranked from the most to least cost-effective in enhancing timeliness and completion of vaccination, RR notification methods are telephone calls, letters, postcards, auto-dialers, and home visits. However, home visits followed by direct telephone calls were the most effective methods for raising vaccine coverage rates.^15^ Mobile phone interventions, including RR notification, effectively improve immunization coverage in low and middle-income countries (LMIC).^16^ This study aimed to evaluate the usage of call back notification in terms of direct phone calls in improving the vaccine coverage rate for children under five in Baghdad province, Iraq, in 2022.

## Methods

This descriptive study evaluated a direct, person-to-person mobile phone re-call intervention that was implemented in Baghdad Province between the 1^st^ of April 1^st^ and the 30^th^ of June 2022. Baghdad, the capital of Iraq, is divided into two local government areas: Karkh, with an under-five population of 532,440 across 126 primary health care centers, and Rusafa, with 650,784 children under five served by 123 centers, each area further organized into ten health districts. Baghdad province was prioritized for this type of intervention since majority of families gave contact information more precisely and monitoring of implementation would be sounder for research.

50 PHCs with the lowest performance regarding coverage rates (defined as less than 80%) were selected, 20 PHCs in 7 districts in Karkh and 30 PHCs in 10 districts in Rusafa. The target population were children under five since their immunization and contact information is already available in the PHCs. Also, they are more vulnerable to acquiring infections and experiencing serious complications. Therefore, children under five who were overdue for one or more of the recommended vaccines in the Expanded Program on Immunization (EPI) schedule, except the BCG vaccine, were included.

Using an official immunization registry and information system, the EPI staff periodically identified children overdue for vaccinations to develop and update a list of potential candidates for call back during the data collection period. The list includes the child’s name, telephone contact information, and vaccine information (due/overdue). Immunization is considered delinquent if, at the time of the call, more than 28 days have passed due to any of the recommended immunizations in the EPI schedule in the first five years of life. If more than one vaccination is overdue at the time of call back, all overdue vaccinations will be accommodated in a single notification. In each PHC, two EPI staff conducted the calls directly from PHCs during working hours. The study excluded children above five years, families without mobile phone access, children already fully vaccinated, and families unwilling to participate (refusal or withdrawal of consent).

A direct, person-to-person telephone recall approach was selected for this intervention to enable interactive communication between EPI staff and caregivers. Evidence from comparable low- and middle-income country settings suggests that telephone call reminders can be more effective than reminders delivered via short message service (SMS) in reminders in improving timely childhood vaccination, particularly by allowing real-time clarification,^17^ confirmation of vaccination status, and engagement with caregivers regarding missed appointments. In contrast, SMS or automated reminder systems are limited to one-way communication and may be less effective in settings where literacy levels vary, phone numbers change frequently, or vaccination records require verification. On this basis, a telephone recall strategy was considered operationally appropriate for integration within routine EPI services in Baghdad Province.

Based on operational capacity and the pilot finding, telephone RR intervention involved six sessions. In each session, one call is made to either a parent of a child or any contact person whose cell phone number has been recorded in the EPI registry logs. The first phone contact was made at the start of the study, and then once biweekly if the child still had not received the vaccination. Follow-up ceased, once the child was brought for vaccination or after the sixth trial during the study period. Parents from PHCs not included in the intervention were not contacted, and routine EPI services continued as usual in those facilities.

A data collection instrument developed specifically for this study was used to document the process and outputs of the recall intervention, capture caregiver responses, and collect immunization data regarding the type and number of overdue and administered vaccine doses. Information recorded included the total of number of phone call attempts; calls answered by parents/caregivers; response types (agreement to schedule an appointment, child already vaccinated, inability to access the PHC, refusal of vaccination, appointment already scheduled, child no longer residing in the household, or caregiver concerns about vaccine safety); and calls not answered (incorrect or changed phone number, disconnected line, busy or unreachable number, calls answered by unauthorized individuals, or hang-ups). The instrument was developed in KoboCollect format and was developed, piloted, and validated by officials from the EPI department prior to implementation.

The intervention was conducted through the PHCs in Baghdad, using standardized recall and follow-up forms. Data were compiled by trained health staff under the supervision of the Directorate of Public Health, ensuring completeness, accuracy, and consistency prior to analysis. A one-day training session was conducted for EPI staff in the selected PHCs on data entry, documentation, and communication skills. The process was piloted in two PHCs, and feedback was considered to correct or change what matters. Number of call sessions, frequency, and timing were subjected to modification after piloting. Vaccine providers had been trained to use a unified form of dialogue as much as possible, considering communication skills. The contact person often knows the context of the population in the catchment area where s/he works and knows how to approach them.

### Ethical considerations

All recall and call-back notifications were conducted in compliance with applicable national regulations on privacy, confidentiality, and data protection. Ethical approval for the study was obtained from the Research Ethics Committee of the Ministry of Health, Iraq, prior to the commencement of data collection. All study procedures adhered to national research ethics regulations and relevant institutional policies. Informed verbal consent was obtained from parents or primary caregivers before participation. Caregivers were provided with a clear explanation of the study’s objectives, procedures, and confidentiality safeguards. The use of verbal consent was reviewed and approved by the Research Ethics Committee and was documented by EPI staff at the time of the call using a standardized study data collection form. Participation was entirely voluntary, and caregivers were informed of their right to withdraw at any time without any consequences for their access to health services.

### Statistical analysis

Descriptive statistical methods were used to analyze the data. Primary outcomes included the number of call attempts, caregiver responses, successful vaccination appointments, and the number and type of vaccines administered. Proportions were calculated relative to total eligible children and overdue doses. Vaccine coverage rates among targeted defaulters were computed. Data was analyzed using Microsoft Excel (version 16), and results were presented in tables and charts.

## Results

**Figure 1.**
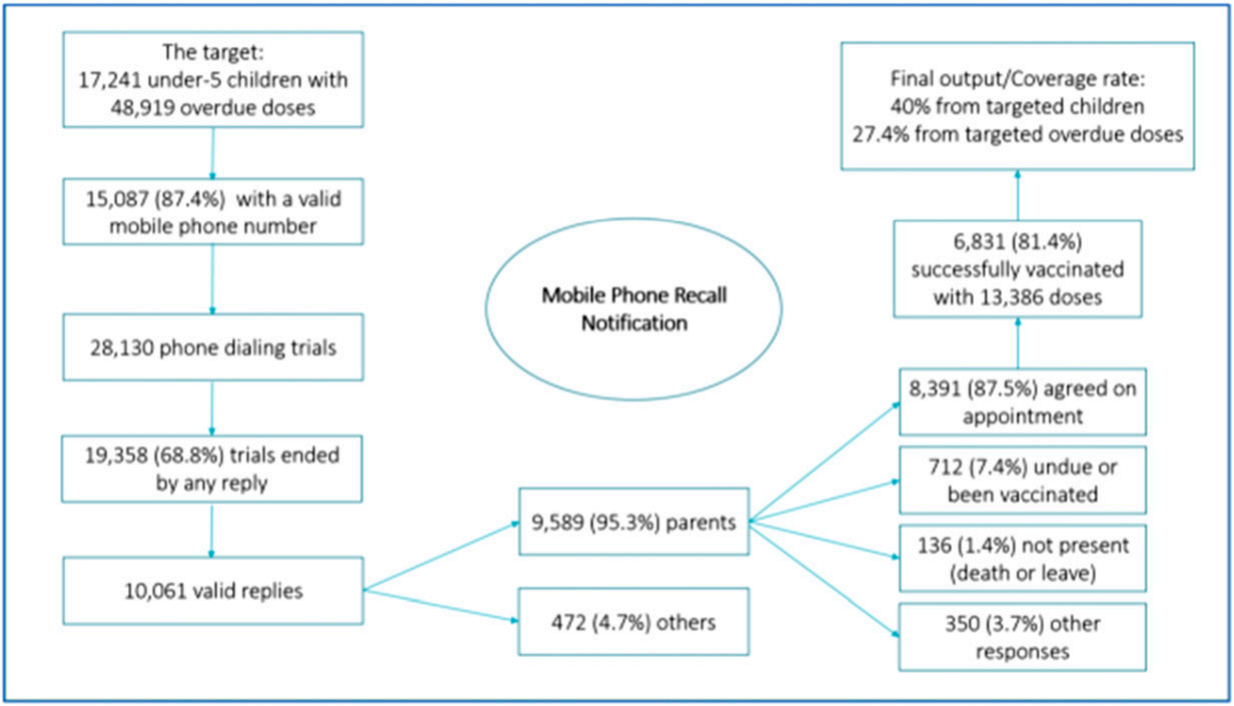
Schematic results of the recall/call back notification, Baghdad 2022.

A total of 17,241 children under five (47.8% females) with 48,919 overdue doses of were identified and targeted in the activity: 25.5% in Karkh and 74.5% in Rusafa (Table 1). Among these children, 15,087 (87.4%) had a valid mobile phone number in the immunization registries. EPI staff conducted 28,130 call attempts, of which 19,358 (68.8%) resulted in 10,061 valid replies, with a mean of 1.9 call attempts per child. These replies corresponded to 66.7% of targeted children with known contact information (Figure 1) (Call attempts were considered complete once the child was brought for vaccination or after the sixth call trial during the study period. Valid reply defines as a reply of anyone on the other side (parents, caregiver, others) whatever the response was.).

**Table 1.** Main output of the recall/call back notification in Baghdad, 2022.

Characteristics	Baghdad-karkh		Baghdad-rusafa		Total	
	No.	%	No.	%	No.	%
Targeted children						
Male	2219	50.5%	6780	52.8%	8999	52.2%
Female	2174	49.5%	6068	47.2%	8242	47.8%
Total children	4393	25.5%	12848	74.5%	17241	100.0%
Estimated overdue doses						
Penta2 (+OPV2, IPV1)	3537	23.2%	11694	76.8%	15231	31.1%
Penta3 (+OPV3, IPV2)	2499	22.3%	8721	77.7%	11220	22.9%
MMR (1 and 2)	2366	28.3%	5985	71.7%	8351	17.1%
Penta1 (+OPV1)	2154	28.0%	5550	72.0%	7704	15.7%
Measles (MCV1)	1336	34.4%	2548	65.6%	3884	7.9%
DTP booster1 (+OPV4)	394	17.6%	1839	82.4%	2233	4.6%
DTP booster2 (+OPV5)	264	89.2%	32	10.8%	296	0.6%
Total	12550	25.7%	36369	74.3%	48919	100%
Not replied						
Line not in service	816	70.7%	2739	70.9%	3555	70.9%
No reply at all	332	28.8%	1077	27.9%	1409	28.1%
The call ended before it was completed	4	0.3%	26	0.7%	30	0.6%
Busy line	2	0.2%	21	0.5%	23	0.5%
Total	1154	23.0%	3863	77.0%	5017	100.0%
Replied						
The child’s parents replied	2050	90.0%	7539	96.9%	9589	95.3%
Someone else replied	229	10.0%	243	3.1%	472	4.7%
Total	2279	22.7%	7782	77.3%	10061	100.0%
Response (from replied parents)						
Agree to make an appointment	1680	82.0%	6711	89.0%	8391	87.5%
Children already vaccinated in the PHC	92	4.5%	339	4.5%	431	4.5%
Other responses	103	5.0%	242	3.2%	345	3.6%
The child is already vaccinated in PHC else	122	6.0%	148	2.0%	270	2.8%
The child no longer lives in the home	36	1.8%	40	0.5%	76	0.8%
Child died	13	0.6%	47	0.6%	60	0.6%
Undue child	1	0.0%	10	0.1%	11	0.1%
Parents Cannot get access to PHC	1	0.0%	2	0.0%	3	0.0%
Parents refused	2	0.1%	0	0.0%	2	0.0%
Parents concerned AEFI	0	0.0%	0	0.0%	0	0.0%
Total	2050	21.4%	7539	78.6%	9589	100.0%
Vaccine doses administered (among those agreed on appointment)						
IPV1	287	8.0%	1010	10.3%	1297	9.7%
OPV2	334	9.4%	961	9.8%	1295	9.7%
Penta2	334	9.4%	957	9.7%	1291	9.6%
OPV3	319	8.9%	956	9.7%	1275	9.5%
Penta3	311	8.7%	961	9.8%	1272	9.5%
MMR1	333	9.3%	896	9.1%	1229	9.2%
IPV2	282	7.9%	941	9.6%	1223	9.1%
Measles	115	3.2%	802	8.2%	917	6.9%
Penta1	210	5.9%	614	6.3%	824	6.2%
OPV1	202	5.7%	615	6.3%	817	6.1%
DTP booster1	272	7.6%	379	3.9%	651	4.9%
MMR2	228	6.4%	213	2.2%	441	3.3%
OPV booster1	0	0.0%	360	3.7%	360	2.7%
DTP booster2	147	4.1%	21	0.2%	168	1.3%
OPV booster2	151	4.2%	13	0.1%	164	1.2%
Rota2	17	0.5%	68	0.7%	85	0.6%
Rota1	26	0.7%	51	0.5%	77	0.6%
Total	3568	26.7%	9818	73.3%	13386	100.0%

**Figure 2.**
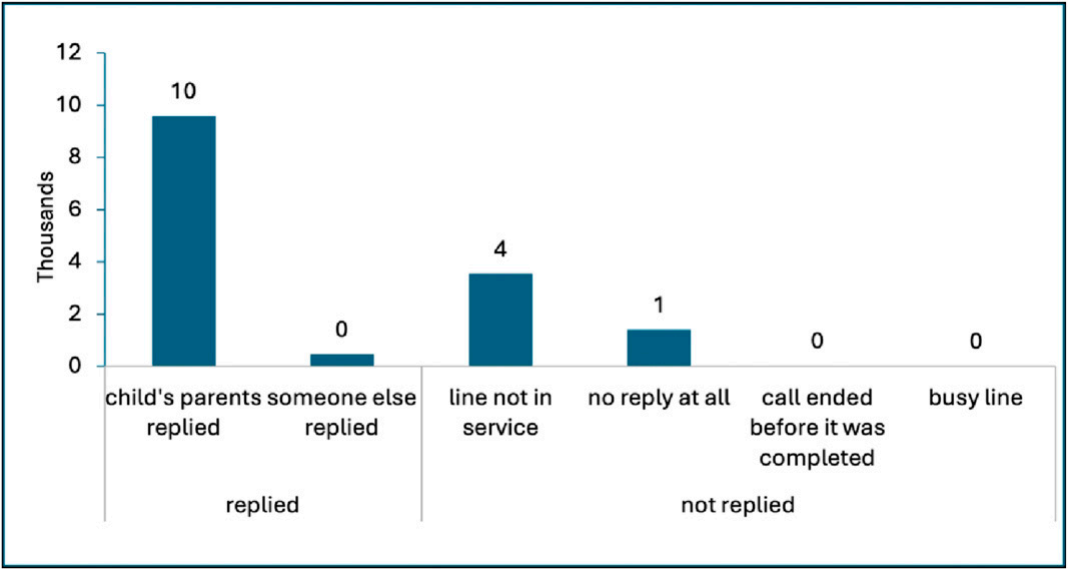
Distribution of replies to recall/call back notification.

**Figure 3.**
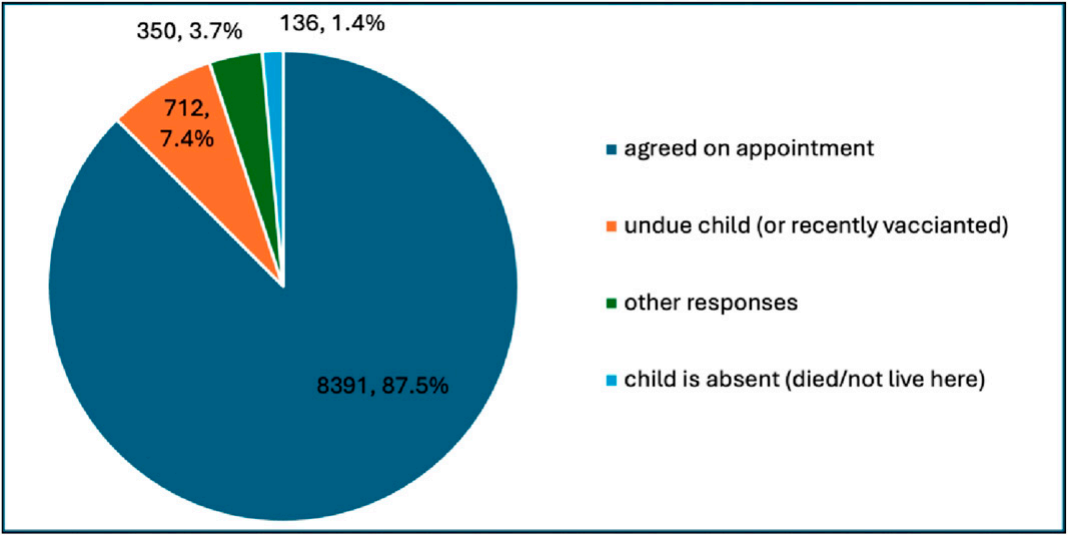
Distribution of responses to recall/call back notification.

Of the valid replies, 9,589 (95.3%) were from parents, while 472 (4.7%) were answered by individuals not related to the targeted children, mainly due to incorrect or changed phone numbers (Figure 2). Among parents who responded, 8,391 (87.5%) agreed to schedule a vaccination appointment for their child. In addition, 712 (7.4%) reported that their child was not overdue or had already been vaccinated at the same PHC or elsewhere, and 136 (1.4%) reported that the child had died or was no longer living with them. Very few parents reported difficulty accessing PHCs (3 responses) or refusal of vaccination (2 responses) (Figure 3).

**Figure 4.**
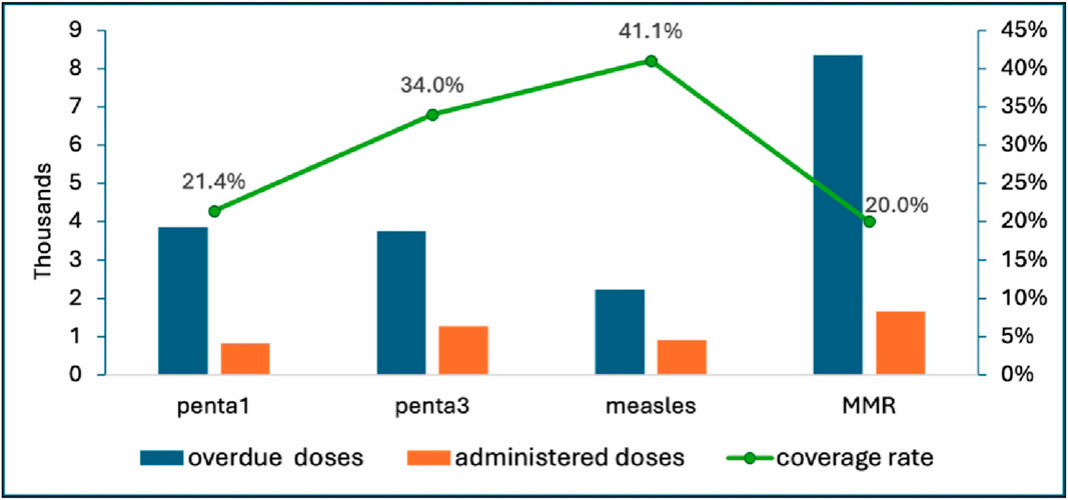
Coverage rates of some vaccines.

Of those appointed for vaccination, 6,831 (81.4%) children attended PHCs during the study period and vaccinated with 13,386 doses of different vaccines, accounting for 39.4% of targeted missed-out children and 27.4% from overall estimated overdue doses (Figure 4). The immunization data were updated by adding the administered doses and correcting the EPI historical data. Of the total doses administered, vaccines were reported according to the national immunization schedule.

Vaccines scheduled at 2 months of age (first recommended doses) were administered at the lowest rates, including Pentavalent-1 (824 doses; 21.4%) and OPV-1 (817 doses; 21%). Vaccines scheduled at 4 months of age (IPV-1, OPV-2, and Pentavalent-2) accounted for the highest number of administered doses (3,883 total doses; 25.5% of corresponding overdue doses). This was followed by vaccines scheduled at 6 months of age (OPV-3, Pentavalent-3, and IPV-2), with 3,770 doses administered (33.6%). Vaccines scheduled after infancy included measles–mumps–rubella (MMR) (1,670 doses; 20%), while measles vaccine alone was administered to 917 children (41%) (Figure 4).

**Figure 5.**
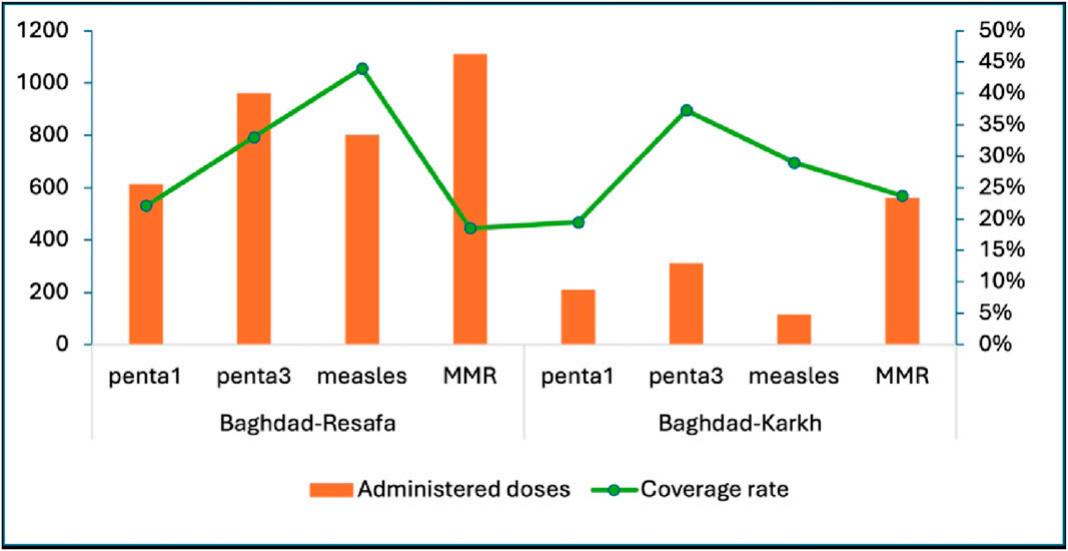
Distribution of coverage rates for some vaccines by region.

In addition, 819 (39.2%) DTP booster doses were administered. The Rotavirus vaccines were the least frequently administered, with only 162 doses recorded (Table 1). Although a higher number of doses were administered in Rusafa than in Karkh, coverage levels were broadly similar between the two areas (Figure 5).

## Discussion

The use of information and communication technologies as interventions to support health systems is a rapidly growing field. However, many lack thorough research on the implementation process, outcome, and health impacts.^18,19^ Our study has shed light on the potential feasibility of cellphone-based call back intervention sessions implemented for missed vaccination appointments in Iraq. Using a list of eligible defaulters, trained vaccine providers in 50 PHCs in Baghdad Province conducted direct telephone calls with caregivers to scheduled new appointments.

Analysis of immunization registries revealed a substantial burden of missed vaccinations, with nearly three-quarters of these defaulters residing in the Baghdad-Rusafa region. Rusafa accounted for the majority of targeted children and overdue doses, consistent with its larger population size and more socioeconomically disadvantaged and overcrowded settings. Despite this higher burden, caregiver engagement with the RR intervention was stronger in Rusafa, as evidenced by higher reply and appointment-acceptance rates compared with Baghdad-Karkh. Rusafa also contributed the majority of administered doses, including a larger number of zero-dose children vaccinated. Taken together, the higher achievements observed in Rusafa appear to reflect both a larger pool of defaulters and stronger caregiver engagement with the RR intervention, resulting in greater absolute vaccination gains compared with Baghdad-Karkh. These findings suggest that RR interventions may yield larger absolute gains in high-burden settings, although operational challenges such as outdated contact information persisted in both areas.

Across both areas, missed doses were more frequent for vaccines scheduled later in the immunization calendar, with measles-containing vaccines contributing a substantial proportion. This pattern is consistent with previous studies showing higher drop-out rates between consecutive vaccines, particularly between pentavalent3 and the first measles-containing vaccine, possibly due to the longer interval separating these doses.^10,11^ These findings underscore the importance of targeted follow-up strategies for children at risk of late-schedule defaulting.

Active outreach through direct telephone contact facilitated re-engagement with many caregivers and supported the conversion of registry-identified defaulters into completed immunizations. This pattern suggests that missed vaccinations in this setting were driven less by outright refusal and more by gaps in follow-up, awareness, or communication. Recall interventions may therefore be most effective as system-strengthening tools that re-engage caregivers already willing to vaccinate, rather than as solutions to entrenched demand-side resistance. The inability to fully meet program targets nonetheless highlights the limits of recall alone and underscores the continued influence of structural constraints and data-quality limitations.

In addition to timing effects, differences in the types of vaccines administered between the two areas suggest variation in immunization continuity and patterns of service contact. The greater contribution of measles-containing vaccines in Rusafa may reflect a higher proportion of children defaulting at later stages of the immunization schedule and subsequently reached through the RR intervention, although differences in population size, service utilization, and baseline coverage likely also influenced this pattern. In contrast, the relatively higher contribution of booster doses in Karkh may indicate stronger early-schedule vaccination uptake, with attrition occurring at later reinforcement visits rather than sustained completion of the full immunization schedule. The consistently low uptake of rotavirus vaccines in both areas highlights persistent challenges in early infancy vaccination, where narrow age-eligibility windows and delayed engagement with services substantially limit opportunities for catch-up immunization. Taken together, these patterns emphasize the importance of earlier, proactive follow-up and sustained continuity of care across the immunization schedule, particularly for vaccines with strict age limitations.

Consistent with these observations, systematic reviews indicate that RR notification systems can improve immunization coverage in primary care settings^20–23^ and increase vaccination uptake in LMIC.^24–26^ However, the overall quality of evidence remains limited, particularly with respect to implementation context, sustainability, and equity effects. Mobile phones nonetheless represent a cost-effective and secure means of delivering public health interventions using information and communication technologies, reinforcing their appeal as tools for strengthening routine immunization systems.^12^

Beyond coverage gains, the intervention yielded important secondary benefits related to data quality. Follow-up communication enabled EPI staff to update immunization records, incorporate newly administered doses, and correct documentation gaps where vaccinations had not been reported to the EPI unit at the corresponding PHC. These improvements highlight the dual function of RR interventions, not only as outreach mechanisms, but also as catalysts for strengthening routine health information systems that underpin immunization performance.

Caregiver responses further suggested that structural barriers such as difficulty accessing PHCs or outright refusal were infrequently cited reasons for missed vaccination. Instead, the call-back process facilitated direct dialogue between vaccinators and caregivers, allowing providers to clarify vaccination status, address concerns, and tailor communication to individual circumstances. This interaction may enhance caregiver awareness and trust in the health system, thereby supporting improved adherence to recommended immunization schedules over time.^27^

At the same time, limitations in immunization registries clearly constrained the efficiency of the RR intervention. A proportion of targeted defaulters lacked documented contact information, primarily caregiver telephone numbers, and even among those with recorded mobile numbers, successful reach was not universal. Unsuccessful contact attempts were most frequently attributable to inactive or out-of-service phone lines and non-response. Moreover, reports from some contacted caregivers that children were not overdue or had been vaccinated elsewhere indicate delays or gaps in updating immunization records. Together, these findings underscore persistent weaknesses in the completeness and timeliness of EPI data, which directly limit the precision and potential impact of recall activities. Consistent with prior evidence, poor data quality remains one of the most frequently cited barriers to effective RR implementation,^20,28^ with similar challenges reported across RR studies that show only modest effect sizes due to difficulties in accurately identifying and contacting eligible families.^29–32^

Beyond data limitations, additional operational constraints affect the scalability and sustainability of RR interventions. Vaccination providers often operate under limited staffing, time constraints, and competing service demands, restricting the routine implementation of recall activities across facilities.^27^ When RR systems are successful, they may also increase workload within immunization rooms, further straining already constrained vaccinator capacity,^33^ particularly in settings where health workers remain heavily engaged in COVID-19 response and vaccine deployment activities.^34^ These financial and human-resource considerations underscore the need to adopt context-appropriate and cost-effective RR strategies that balance potential coverage gains with system capacity and long-term sustainability.^15^

## Strengths and limitations

This study demonstrates the feasibility and effectiveness of a low-cost, direct, person-to-person phone recall intervention to improve routine childhood immunization in an urban setting in Iraq. Key strengths include its integration within existing EPIEPI infrastructure, relatively straightforward implementation, and standardized training provided to staff, which supported consistency in communication and reporting. The intervention was applied across two large administrative zones and involved a sizeable, well-defined target population, enhancing both statistical power and local relevance. In addition to improving vaccine uptake, the intervention exposed systemic challenges such as incomplete or outdated contact information and enabled immediate improvements in immunization registry accuracy, representing an important secondary programmatic benefit.

However, several limitations should be considered. First, the study was conducted in PHCs selected for very poor baseline immunization performance and did not include a control group. Because facilities were chosen based on extreme underperformance, part of the observed improvement may reflect regression to the mean rather than the effect of the intervention itself. In addition, the study did not include direct attribution measures, such as asking caregivers whether the phone call was the primary reason for attending the PHC. Consequently, it is not possible to determine with certainty the extent to which vaccination uptake was directly caused by the recall intervention, and findings should be interpreted as descriptive and associative rather than causal.

Second, the intervention depended on the availability of valid phone numbers, resulting in the systematic exclusion of families without mobile access or with inaccurate registry data. This introduces reachability bias and raises equity concerns, as children most at risk of missing vaccination were also the least likely to be contacted through phone-based methods. Attendance at PHCs and vaccine administration outcomes were based on PHC immunization records, while self-reported parental responses were used primarily to capture reasons for non-attendance or claims of prior vaccination. Third, some outcome information relied on parental self-report, which may be subject to recall errors, misreporting, or social desirability bias. The relatively short three-month follow-up period further limits interpretation, as it captured only immediate vaccination uptake and did not allow assessment of longer-term adherence, schedule completion, or sustainability of coverage gains.

Additional limitations include potential variation in how consistently the recall protocol was applied across PHCs, the absence of cost or workload assessments to inform scalability, and the influence of external factors such as seasonal patterns, population mobility, and competing health priorities during the study period.

## Implications for future research

Future research should adopt more robust study designs, such as controlled or quasi experimental trials, to strengthen causal inference and avoid attributing observed improvements solely to recall interventions. Comparative studies are needed to assess the relative effectiveness of different reminder and recall modalities, including SMS, automated calls, messaging applications, and community health worker outreach, as well as combinations of these approaches. Importantly, future evaluations should move beyond immediate vaccination uptake to examine outcomes such as timeliness, schedule completion, and reduction in vaccine drop out across the immunization course.

Equally important is the systematic assessment of cost effectiveness and scalability, particularly with respect to staff time, workload implications, and resource requirements associated with person-to-person telephone calls compared with more automated strategies. Qualitative research should further explore reasons for persistent non reachability or refusal, incorporating perspectives from both caregivers and vaccinators to support the development of more context sensitive, equitable, and acceptable approaches. Finally, future studies should test strategies to improve and maintain accurate contact and immunization data through strengthened registries and assess whether the benefits of recall interventions are sustained over time and lead to lasting improvements in immunization program performance.

## Conclusion

This study shows that cellphone re-call notifications are a feasible and effective way to improve routine childhood immunization in PHC settings with high mobile phone coverage, while also highlighting the importance of maintaining accurate contact information and reliable registry data. As a practical, low-cost intervention embedded within existing health system structures, it demonstrates the potential of digital tools to close gaps in coverage and to strengthen program performance. Beyond immediate gains, this work provides a model for how immunization services in Iraq could adopt re-call as part of a national tracking and reminder system to systematically reach children who are overdue for vaccination. To move from proof of concept to sustained impact, however, further research is needed to quantify effect sizes more rigorously, assess cost-effectiveness and scalability in both urban and rural settings, and determine how best to integrate re-call with other reminder modalities and health worker outreach. By addressing these questions, re-call interventions could form a cornerstone of strategies to achieve higher and more equitable vaccine coverage across Iraq.
